# An Improvement in the Reliability of Standard Cell Enclosures

**DOI:** 10.6028/jres.093.144

**Published:** 1988-08-01

**Authors:** Bruce F. Field, Liu Ruimin

**Affiliations:** National Bureau of Standards Gaithersburg, MD 20899

**Keywords:** reliable temperature controller, standard cell enclosure, temperature protection circuit, temperature-regulated oven

## Abstract

We describe the design of a new temperature-regulation circuit, which is used as an outer oven controller for new standard cell enclosures, with the emphasis on improving the reliability of the temperature control. A redundant protection circuit is used to prevent loss of temperature control caused by component failures in the controller. The temperature control of the outer oven of the enclosure is better than 0.4 mK per °C change in ambient temperature. When used with the additional inner controller the sensitivity of the cell temperature to the ambient temperature is improved to 20 *μ*K/°C. This paper describes in detail the new circuit, summarizes the enclosure construction, and presents data on the performance of the system.

## 1. Introduction

If saturated standard cells are to be used as a high precision voltage reference, they must be kept in a constant temperature-regulated oil bath or air enclosure because of their large temperature coefficient of EMF. The requirements for such an enclosure are good temperature stability and high reliability of the temperature control circuit. The importance of the reliability requirement is often overlooked. If the enclosure (and cell) temperature changes significantly (several degrees Celsius), the chemical equilibrium within the cell is disturbed and after control is re-established it may still take several weeks or months for the cell EMFs to recover to their original values. Sometimes an unpredictable permanent change in the cell EMFs can also occur. Recently, two new standard cell enclosures have been constructed with the emphasis on reliability of the temperature control circuitry. The new enclosures are similar to two older standard cell enclosures, previously constructed at NBS in 1974, but the controller circuitry has been redesigned to be more reliable and to replace obsolete components [1]. One problem has been that the extensive protection circuits used in the original enclosures contain several components that fail routinely. The concern for improved reliability has led to the development of redundant protection circuits in the new enclosures to prevent loss of temperature control caused by component failures in the controller.

The mechanical construction details of the new enclosures are the same as for the previous enclosures, i.e., they consist of four concentric aluminum cylinders separated by formed polystyrene insulation with two of the four cylinders controlled by an outer oven controller and an inner oven controller. As in the original design, the outer cylinder is unheated and thermally lags the enclosure from ambient temperature changes. The next two cylinders are the outer heater and inner heater, respectively, with the last cylinder being a temperature lagged cell compartment to further reduce any temperature gradients. However, the original controller circuitry has been changed completely. A simple control circuit using a platinum resistance thermometer with chopped-dc excitation and phase sensitive detection developed by Cutkosky and Davis is used as the inner oven controller [2]. The outer oven controller contains a main controller, a backup controller, and an arbitration circuit that connects the outer oven heater to the main controller or to the backup controller in the event of main controller failure. This paper describes the outer oven controller and protection circuitry and presents data on the performance of the system.

## 2. Circuit Details

The enclosure is designed for an inner oven temperature of 30°C with the outer oven sėt to 29.9 °C. Thus, the outer oven provides most of the power, 4 W with an ambient temperature of 23°C, while the inner oven typically supplies 75 mW with a maximum output capacity of 300 mW. Because of the limited power capacity of the inner oven (and the correspondingly limited temperature increase), guaranteeing the integrity of the outer oven is sufficient to prevent extreme changes in the inner oven temperature even in the event of an inner oven failure.

### 2.1 Outer Oven Controller

**Figure 1:**
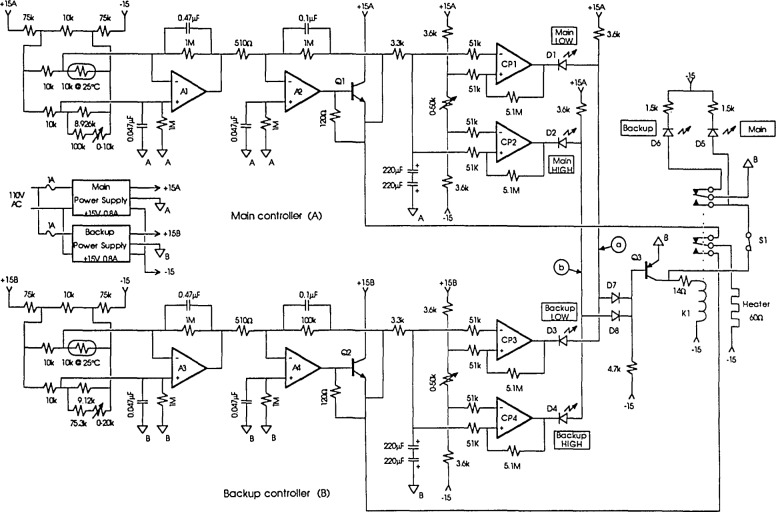
Circuit diagram of the outer oven controller.

The main controller and backup controller for the outer oven are nearly identical control circuits using independent thermistor sensors in Wheatstone bridges and high-gain dc amplifiers to provide proportional temperature control (see fig. 1). Power to each controller is supplied by its own independent well-regulated power supply. The outer oven temperature (for each controller) is monitored by a thermistor (10 kΩ @ 25°C) mounted in a small hole drilled in the enclosure and filled with silicon heat sink grease. The thermistor forms one arm of a nearly equal-arm Wheatstone bridge powered by a low-voltage derived from the regulated power supplies. The output of the bridge is amplified by a two-stage low-offset-voltage dc amplifier which in turn drives a transistor to boost the output for the 60-Ω heater.

The arbitration circuit consists of four voltage comparators, a transistor and a sensitivity relay. This circuit determines which controller is operating correctly and switches the proper controller output to the outer oven heater. Each controller output is compared to a high and low limit using two comparators. The high and low limits are set to ± 10 V for the main controller and ± 5 V for the backup controller. The outputs of the controllers exceed either of the limits only if a controller has failed or the enclosure is warming up from ambient temperature. Under normal conditions when the controller outputs are within the limits, the output voltages of the four comparators CP1–CP4 are high (see fig. 1). The four diodes, D1–D4, are cut off and the potentials of points a and b are high, turning on diodes D7 and D8 and forcing the transistor Q3 to be turned off and the relay to be released. The main controller output is thus connected to the outer oven heater. This arrangement extends the life of the relay and transistor Q3 because they are normally not operated.

If the main controller were to fail due to a faulty component and supply no power to the heater while the backup controller continued operating properly, the output of comparator CP2 would go low and diode D2 would turn on. As the temperature of the enclosure drifted down eventually CP3 would go low turning on diode D3, forcing potentials a and b low, and causing the relay to operate. A similar sequence occurs if the main controller were to fail and supply full power to the heater. The logic requires that both controllers indicate that the enclosure temperature is out of control and to disagree as to the direction (high or low) before switching to the backup controller. This permits the enclosure to warm from room temperature without switching to the backup controller. Once the backup controller has gained control the second set of contacts on the relay provide a self-locking arrangement to keep the relay energized. The control can be returned to the main controller only by opening switch S1 manually. A failure of the backup controller has no effect on the operation of the circuit as long as the main controller operates normally. Table 1 summarizes all the failure conditions and the status of the relay.

Diodes D1–D6 are light emitting diodes mounted on the front panel of the controller to indicate the status of the outer oven temperature control. One of the advantages of this design is that a failure of the backup controller can be discovered immediately by observing the light emitting diodes, even if it is not the active controller.

The main and backup controllers differ only with respect to the total circuit gain, with gains of 2.17×10^5^ and 2.17×10^4^, respectively. The gain of the main controller was empirically determined for good regulation and rapid recovery from ambient temperature changes. The gain of the backup controller was deliberately set lower to avoid a conflict between the controllers due to their temperature set points drifting apart. It should be noted that for proper operation the control temperatures (set points) of the main controller and backup controller must be carefully adjusted to the same value. This was accomplished when the enclosures were first put under test by adjusting the trim resistor on the backup controller, which parallels an arm of the bridge, to equalize the outputs of the controllers. Under normal operating conditions a change in the set point of the main controller of 5 m °C (due to a drifting resistor in the bridge for example) would cause a negligible change in the amplifier output to the heater, while the voltage output of the backup controller would change 1V. With the currently set limits of the comparators the two controller set points can drift apart as much as 0.025 °C (and this is considered to be an unlikely occurence) before a failure is indicated.

### 2.2 Inner Oven Controller

**Figure 2:**
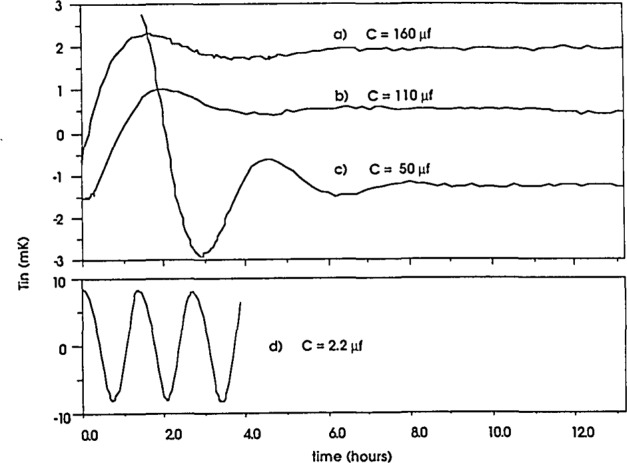
Response of the inner oven controller to a temperature perturbation for various values of feedback capacitance. Curves a–c are from one enclosure, d is from the second enclosure.

The inner oven is held at a temperature of 30°C, approximately 0.1 °C higher than the surrounding outer oven. The heater voltage is indicated on a meter mounted on the front of the controller and the outer oven temperature is adjusted so the inner oven output voltage is at mid-scale (≈75 mW which corresponds to a 0.1 °C temperature rise). A platinum resistance thermometer (110 Ω @ 30°C) was chosen as the sensor because of its excellent long-term stability, although its temperature coefficient is much smaller than that of the thermistor and the output of the bridge is only 63.3 nV/mK. In order to avoid the effects of dc voltage offsets and drifts in a dc amplifier circuit, a circuit consisting of a chopped dc amplifier and a phase sensitive detector was chosen [2]. Its output is used to drive a 3000 Ω-heater via a current booster. An additional power supply, separate from the ones used for the outer ovens, is used to power the circuitry. It has been proven experimentally that the circuit will meet our requirement for high-stability temperature control with day-to-day variations of less than 50 *μ*K. Since the enclosure is a system with large thermal inertia, the time constant of the controller feedback must match it. Figure 2 shows the temperature of the inner controller in one of the enclosures in response to a perturbation with different values of capacitance in the feedback loop. From these curves we calculated an optimum value for the capacitance for fast recovery and long term stability.

## 3. Performance

**Figure 3:**
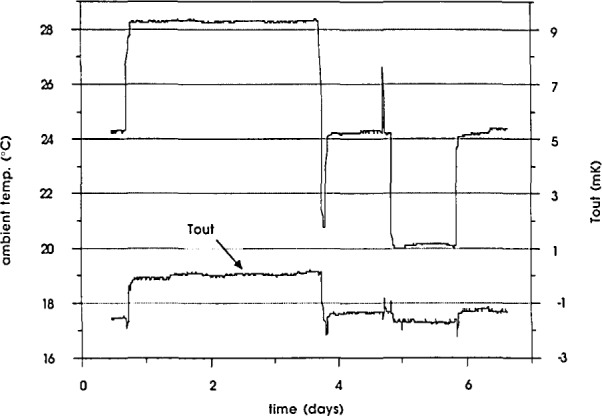
Outer oven temperature change with the ambient temperature for one of the enclosures.

In order to test the stability of temperature control of the enclosures with a changing ambient temperature, the enclosures were placed in a temperature-controlled chamber and the temperature was changed in the sequence 24°C, 28°C, 24°C, 20°C, 24°C. The enclosures were held at each temperature for one or two days. The outer oven temperature was monitored by measuring the output of the backup controller. A plot of the outer oven temperature change with ambient temperature for one of the enclosures is shown in figure 3. The change of the outer oven temperature with the ambient temperature is not linear because it is proportional to the square of the output voltage of the outer oven controller. The result is that the variation of the outer oven temperature is less than 400 *μ*K for ambient temperature changes of 1 °C. The maximum temperature excursion over a period of more than fifty days was found to be less than 1 mK.

**Figure 4:**
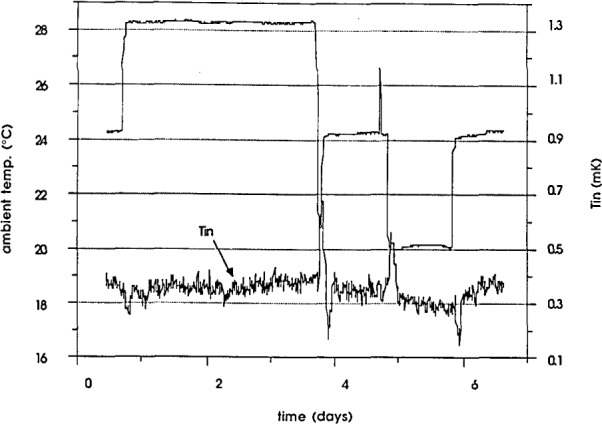
Variation of the cell compartment temperature with the ambient temperature in one of the enclosures.

The temperature of the cell compartment of the enclosure is monitored by two thermistors mounted in a hole in the bottom of the compartment. The thermistors (10 kΩ @ 25°C) are connected in opposite arms of a Wheatstone bridge with two low-temperature-coefficient resistors (10 kΩ) in the other arms. The low-temperature-coefficient resistors are contained within the outer oven to further improve their stability. The bridge is powered from the regulated power supply of the inner oven. The output of the bridge is 4.155 mV / °C with only 1.37 *μ*W dissipated in each thermistor. The output is measured directly by a nanovoltmeter or a high-resolution DVM. For the following measurements a DVM was used that had a short term measurement noise of 0.02 *μ*V, which corresponds to 5 *μ*K. As shown in figure 4 the variations of the cell compartment temperature as measured by this thermistor bridge are less than 20 *μ*K for ambient temperature changes of 1 °C.

## 4. Conclusions

We have demonstrated that with a small amount of additional circuitry a substantial improvement can be made in the reliability of a temperature controller. With this design, a failure of anyone component will not cause a significant aberration to the cell temperature and a failure can be detected immediately so that corrective action can be taken. In addition, the circuitry has been simplified over the original design, thus greatly reducing the possibility of a failure. Despite the simplicity of the outer oven controller it can be used alone to obtain temperature control with a stability of 1 mK or, as shown here, it can be used with an additional inner controller to provide even better stability.

## Figures and Tables

**Table 1:** Operating Conditions and Status of the Outer Oven Controller

Output of		Output of Comparators				Potential of		Relay Condition	Status of Controller
A	B	1	2	3	4	a	b		
Normal	Normal	1	1	1	1	1	1	released	A, B operating normally
Normal	Low	1	1	1	0	0	1	released	B failure
Normal	High	1	1	0	1	1	0	released	B failure
Low	Normal	1	0	1	1	0	1	released	Probably warming from ambient temp. if A fails, B will eventually go high
High	Normal	0	1	1	1	1	0	released	Probable A failure, B will eventually go low
Low	Low	1	0	1	0	0	1	released	Warming from ambient temp.
High	High	0	1	0	1	1	0	released	Ambient temp. exceeds control temp.
Low	High	1	0	0	1	0	0	operated	A fails, control transferred to B
High	Low	0	1	1	0	0	0	operated	A fails, control transferred to B

A ---- Main controller. B ---- Backup controller. 1 ---- High level voltage. 0 ---- Low level voltage.
